# Métastase ombilicale d'une tumeur rectale : à propos d'un cas

**DOI:** 10.11604/pamj.2014.17.38.3671

**Published:** 2014-01-20

**Authors:** Issam Yazough, Mohammed Amine el Maati Allah

**Affiliations:** 1 Faculté de Médecine et de Pharmacie de Fès, Université Sidi Mohammed Ben Abdellah, Département de Chirurgie, CHU Hassan II Fès, Route de Sidi Hrazem, Maroc

**Keywords:** Metastase, ombilic, tumeur rectale, metastasis, umbilicus, rectal tumor

## Images in medicine

Les métastases ombilicales des tumeurs viscérales sont rares. Connues sous le pseudonyme de nodule de Marie Joseph, elles constituent parfois le signe d'appel de la maladie cancéreuse. Nous rapportons le cas d'un patient âgé de 28 ans, sans antécédents pathologiques notables, qui présentait syndrome rectale. A l'examen physique, le patient avait un nodule ombilicale et au toucher rectal un processus à 6 cm de la marge anale. La biopsie de la tumeur était en faveur d'un adénocarcinome moyennement différencié. Une biopsie exérèse du nodule ombilicale a été réalisée et l’étude anatomopathologique est revenue en faveur d'une localisation secondaire d'un adénocarcinome. Le patient a été adressé en oncologie pour éventuelle radiochimiothérapie concomitante avant la chirurgie. Les métastases ombilicales des tumeurs viscérales demeurent rares; en comparaison avec les métastases hépatiques, pulmonaires, péritonéales, et autres. Elles se voient dans environ 3 à 4% des tumeurs malignes. Cette rareté paraît liée à la difficulté d'envahissement de “l'organe”. En effet, les différentes voie de dissémination des cellules néoplasiques vers l'ombilic sont: la dissémination hématogène, l'extension depuis le ligament rond hépatique jusqu'au ligament ombilical moyen de l'ouraque, par contiguïté à partir de la surface antérieure du péritoine à travers les vaisseaux dermiques de l'ombilic, enfin par un mécanisme de reflux lymphatique rétrograde. Les métastases ombilicales des tumeurs viscérales sont rares. La recherche de la tumeur primitive n'est pas toujours aisée malgré les progrès techniques actuels.

**Figure 1:**
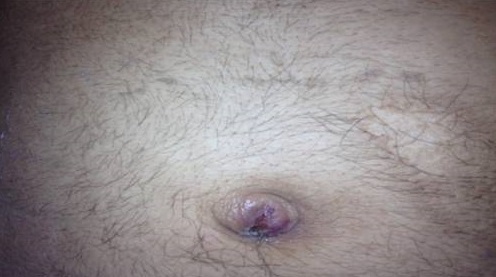
Métastase ombilicale

